# Identifying DNA methylation in a nanochannel

**DOI:** 10.1080/14686996.2016.1223516

**Published:** 2016-10-11

**Authors:** Xiaoyin Sun, Takao Yasui, Takeshi Yanagida, Noritada Kaji, Sakon Rahong, Masaki Kanai, Kazuki Nagashima, Tomoji Kawai, Yoshinobu Baba

**Affiliations:** ^a^ Department of Applied Chemistry, Graduate School of Engineering, Nagoya University, Nagoya, Japan; ^b^ ImPACT Research Center for Advanced Nanobiodevices, Nagoya University, Nagoya, Japan; ^c^ Japan Science and Technology Agency (JST), PRESTO, Saitama, Japan; ^d^ Institute of Materials Chemistry and Engineering, Kyushu University, Fukuoka, Japan; ^e^ Institute of Scientific and Industrial Research, Osaka University, Osaka, Japan; ^f^ Health Research Institute, National Institute of Advanced Industrial Science and Technology (AIST), Takamatsu, Japan

**Keywords:** DNA methylation, single DNA molecule, nanochannel, DNA contraction, 30 Bio-inspired and biomedical materials, 102 Porous / Nanoporous / Nanostructured materials

## Abstract

DNA methylation is a stable epigenetic modification, which is well known to be involved in gene expression regulation. In general, however, analyzing DNA methylation requires rather time consuming processes (24–96 h) via DNA replication and protein modification. Here we demonstrate a methodology to analyze DNA methylation at a single DNA molecule level without any protein modifications by measuring the contracted length and relaxation time of DNA within a nanochannel. Our methodology is based on the fact that methylation makes DNA molecules stiffer, resulting in a longer contracted length and a longer relaxation time (a slower contraction rate). The present methodology offers a promising way to identify DNA methylation without any protein modification at a single DNA molecule level within 2 h.

## Graphical abstract

**Figure:**
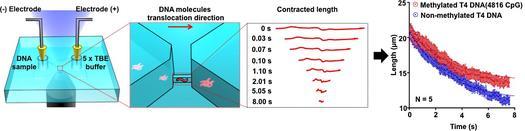

## 1. Introduction

DNA methylation is a stable epigenetic modification that it is known to be involved in the regulation of gene expression. Researchers have reported DNA methylation in eukaryotic cells might lead to silencing of important genes, such as tumor suppressor genes.[1] This gene silencing for tumor suppressor will affect related transcriptional pathways, and ultimately lead to cancer progression.[2] To date, methylation for the promoter regions of tumor suppressor genes have been detected in patients with lung, hepatic, breast, cervical, colorectal and genitourinary cancers.[3] In addition, the methylated number at single DNA molecules has been found to increase abnormally in breast and prostate cancers.[4] In order to detect and predict various cancers in their early stage, simple and rapid detection of methylated DNA molecules at a single molecule level is desired.

**Figure 1.**
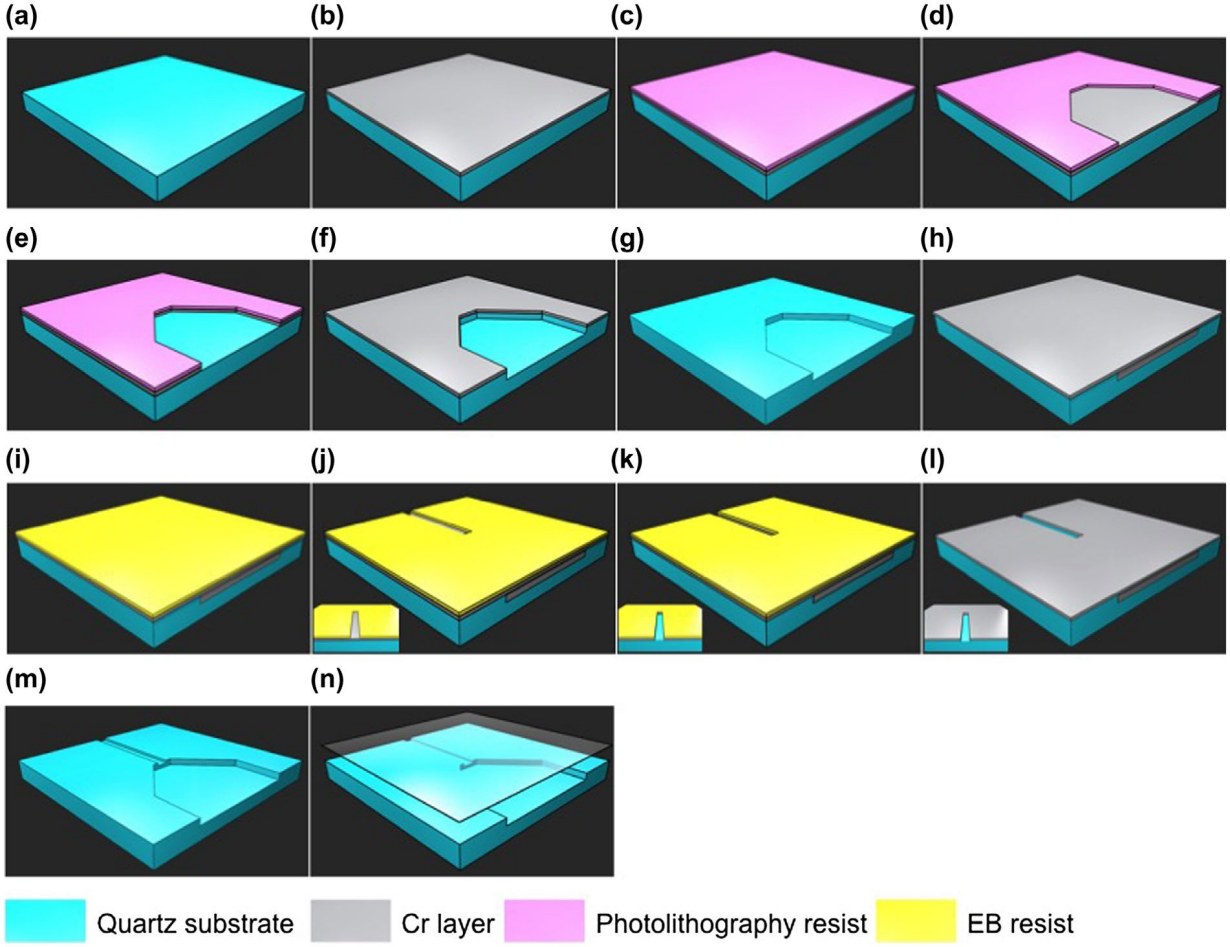
The multi-step fabrication process of the nanochannel device. (a) Cleaning quartz substrates; (b) depositing a Cr layer; (c) coating photolithography resists; (d) drawing microchannel patterns; (e) etching the Cr layer; (f) etching the microchannels; (g) etching the remaining Cr layer; (h) deposition of another Cr layer; (i) coating EB resist; (j) drawing nanochannels; (k) etching the second Cr layer; (l) etching the nanochannels; (m) etching the remaining second Cr layer; (n) bonding with a cover glass.

**Figure 2.**
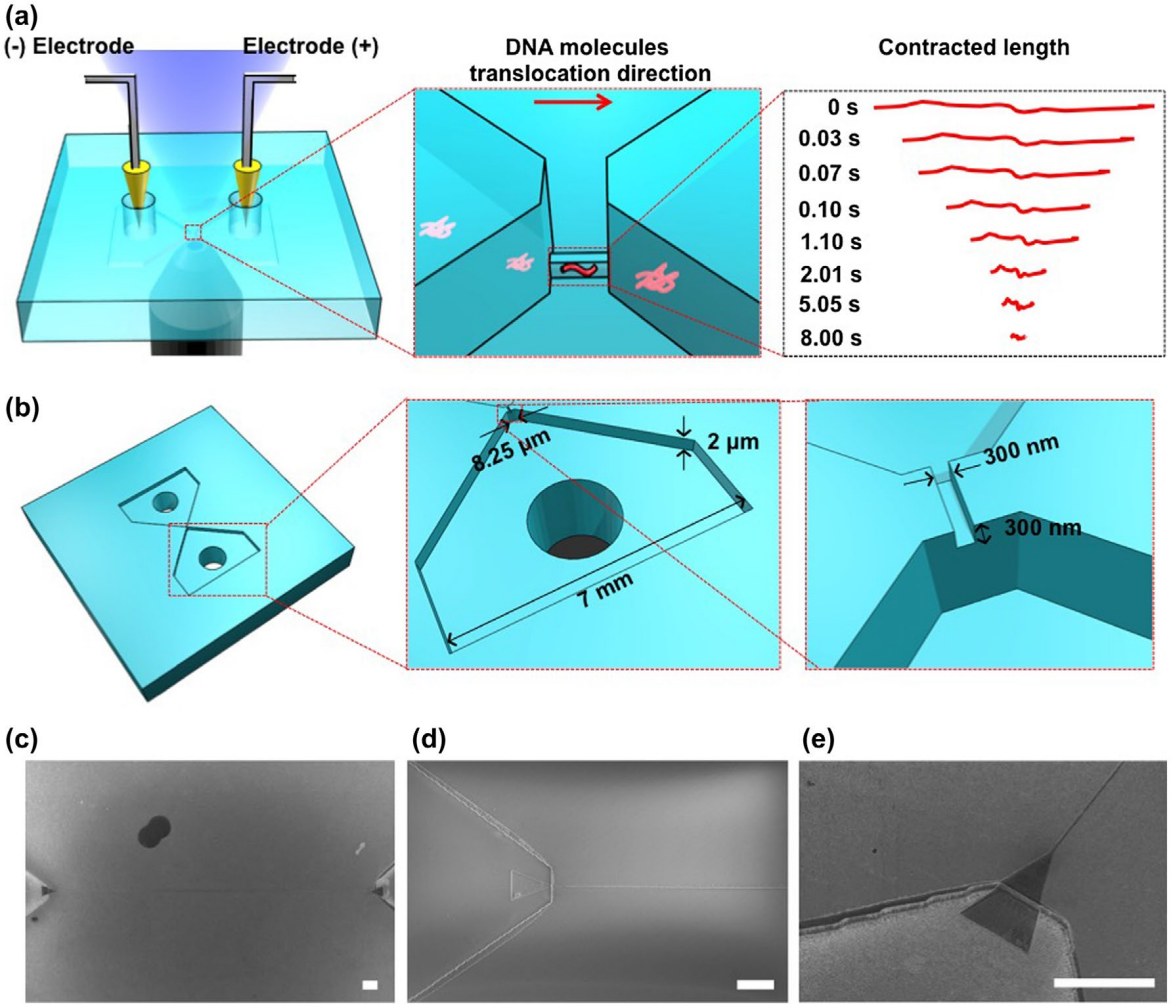
Methylation detection system using the nanochannel device. (a) Schematic illustration of the experimental setup. Negatively charged DNA molecules were transported into a nanochannel by applying an electric field. The red arrow shows the translocation direction of the DNA molecules in the nanochannel. Contracted length analysis of DNA molecules is shown on the right. The contracted length of a single DNA molecule was observed with an inverted fluorescence microscope after switching off the electric field. (b) Schematic illustration of the fabricated device. The device has a nanochannel connected to two microchannels. (c) Scanning electron microscope (SEM) image of the nanochannel with a 300 nm width, 300 nm depth and 250 μm length; scale bar, 10 μm. (d) SEM image of the nanochannel entrance; scale bar, 10 μm. (e) SEM image (30° tilt angle) of the nanochannel entrance; scale bar, 10 μm.

The best-known detection methods for DNA methylation are bisulfite conversion followed by sequencing [5] and chromatin immunoprecipitation followed by sequencing.[6] These strategies can obtain details of the epigenetic modification; however, they have serious disadvantages of consuming a relatively large sample and taking much time. Micro- and nano-devices are powerful tools that have been applied to analyze DNA methylation. They allowed researchers to analyze DNA methylation through optical and electrical approaches by detecting DNA molecules with methylation on the methyl-CpG-binding domain (MBD).[7,8] However, DNA methylation detection in previous studies is based on modification of methylation sites using MBD proteins, which has a risk of non-specific absorption to DNA molecules. Such a non-specific absorption is crucial for the detection of DNA methylation, and therefore a simple DNA methylation detection without any modification is desired for DNA methylation detection with no site specificity. Here we fabricated a nanochannel device to detect the DNA methylation without site specificity at a single DNA molecule level by measuring a contraction process (Figure 1). The nanochannel device was fabricated on fused silica substrates by photolithography, reactive ion etching and electron beam lithography techniques. To measure the contraction process, we introduced methylated and non-methylated single DNA molecules into the nanochannel to elongate and contract them (Figure 2(a)). Both ends of the nanochannel were connected to microchannels, which have reservoirs to put electrodes for introduction of the DNA molecules (Figure 2(b–e)). The nanochannel was 300 nm, 300 nm, and 250 μm in width, height, and length, respectively. The microchannels were 2 μm in height and 5 mm in length, and the widths of the microchannels were gradually changed from 8.25 μm to 7 mm. As we applied electrical potential difference of 3 V between two reservoirs, negatively charged DNA molecules were driven from a reservoir to the other one via the microchannels and the nanochannel, electrophoretically. If the nanochannel geometry is smaller than the gyration radius of the DNA molecules, the DNA molecules were forced to deform from random-coil to linear conformation inside the nanochannel, and finally, the DNA molecules had elongated conformation. After elongation of DNA molecules, we turned off the electrical potential to evaluate the contraction process of DNA molecules with and without methylation inside the nanochannel. This simple analysis method helps to detect DNA molecules with methylation.

## 2. Experimental details

### 2.1. Device fabrication

At first, in order to pattern the microchannel, a Cr layer as a metal mask was deposited on a quartz glass substrate using a sputtering apparatus (model SVC-700LRF, Sanyu Electron Co., Tokyo, Japan). Then, a positive photoresist (TSMR-V50 EL, Tokyo Ohka Kogyo Co. Ltd, Tokyo, Japan) was spin-coated on the Cr layer. After that, the microchannel pattern was developed using AZ 300 MIF developer (AZ Electronic Materials Plt, Tokyo, Japan). Next, the Cr layer in the microchannel pattern was removed by immersing the substrate in Cr etchant solution. The microchannel was etched to a 2 μm depth by reactive ion etching (RIE; RIE-10NR, Samco Co., Kyoto, Japan). The remaining Cr layer was lifted off using Cr etchant solution. Then, another Cr layer of 20 nm thickness was deposited on the quartz glass substrate by rf sputtering for the nanochannel fabrication. The positive electron beam (EB) resist (ZEP520A-7, Zeon Corp., Tokyo, Japan) was spin-coated on the sputter-deposited Cr layer. The nanochannel pattern was formed by electron beam lithography (EBL; SPG-724, Sanyu Electron Co.), and developed using electron beam developer solution (ZED-N50, Zeon Corp.). The second Cr layer in the nanochannel pattern was lifted off using Cr etchant solution. After that, the 300 nm nanochannel depth was etched by RIE. The remaining Cr layer was removed using Cr etchant solution. Finally, the nanochannel device was bonded using a cover glass (Crystal Base Co., Osaka, Japan) by a chemical bonding method.

### 2.2. Sample preparation

T4 DNA molecules (166 kbp, T4GT7 DNA, Nippon Gene Co. Ltd, Tokyo, Japan) were used for identifying occurrence of DNA methylation in the nanochannel. The non-methylated and methylated T4 DNA molecules were stained with an intercalating fluorescence dye YOYO-1 at a dye-to base pair ratio of 1:5. For a single DNA molecule observation, the T4 DNA solution was diluted to 5 ng ml^–1^. In order to prepare the methylated T4 DNA molecules, a mixture of phage T4 DNA molecules (166 kbp, T4GT7 DNA, Nippon Gene Co., Ltd) and CpG methyltransferase (M.SssI) (New England Biolabs Inc., Tokyo, Japan) was incubated at 37°C for 1 h, and then heated at 65°C for 20 min to inactivate the methyltransferase. The CpG methyltransferase (M.SssI) methylates all cytosine residues (C^5^) within the double-stranded dinucleotide recognition sequence 5′...CG...3′. The M.SssI could methylate all 4816 CpGs in the T4 DNA molecules, which are distributed throughout the entire molecule. After that, the methylation state was confirmed using the restriction enzyme SalI (New England Biolabs Inc.), which can digest DNA molecules in the methylation region. The methylated T4 DNA molecules were stained by YOYO-1 overnight at 4°C before use.

### 2.3. Measurement setup

T4 DNA molecules were introduced into the nanochannel under an applied DC electric field provided by a high voltage source-measure unit (model 236, Keithley, Cleveland, USA). Stretching of the T4 DNA molecules was observed and was attributed to the electric force pulling them into the nanochannel against the entropic barrier at the entrance of the nanochannel. Since electrical potential difference less than 3 V between two reservoirs could not transport DNA molecules from microchannel to nanochannel due to the entropy barrier at the entrance of nanochannel, we used 3 V as electrical potential difference, which took 20 min to transport the DNA molecules from microchannel to nanochannel. When the T4 DNA molecules were fully inside the nanochannel, the electric field was switched off immediately. Then, the contraction process of the T4 DNA molecules was observed. We measured nine DNA molecules, four of which showed relatively short DNA length due to photocleavage of DNA molecules; therefore we used only five DNA molecules data (successful rate: 55%). The fluorescence images of T4 DNA molecules were obtained with an inverted fluorescence microscope (ECLIPSE TE300, Nikon, Tokyo, Japan) coupled with a CCD camera (C7190-43, Hamamatsu Photonics K.K.) through a 100 × / 1.40 NA objective lens. A 488 nm laser (FLS-448-20, Sigma Koki Co., Ltd.) was used as a light source to observe the fluorescently stained T4 DNA molecules. The fluorescence images were recorded on a DV tape (Sony DV 180 ME Digital Video Cassette, Sony Corp.).

## 3. Results and discussion

**Figure 3.**
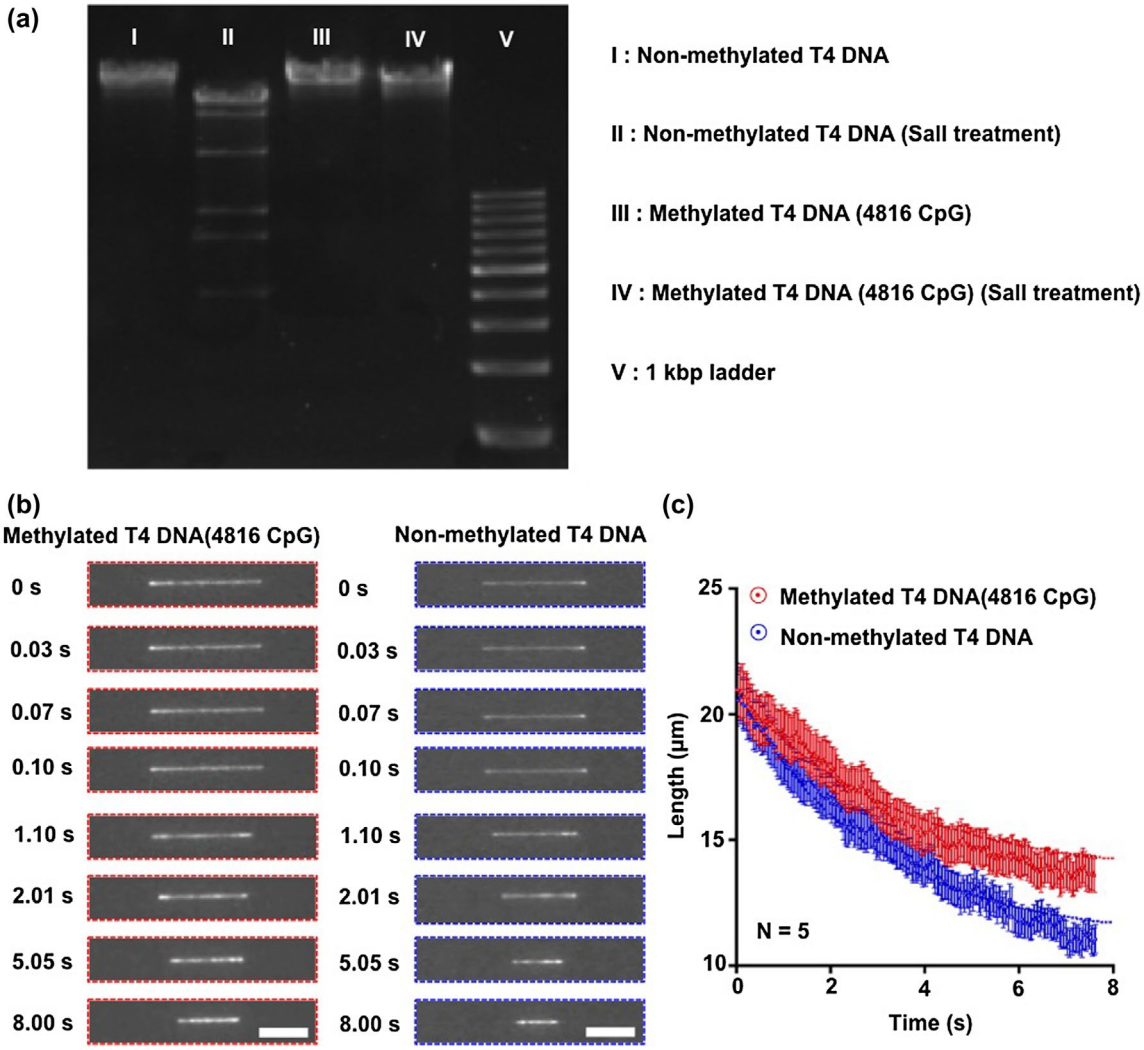
Contracted length analysis of T4 DNA molecules in the nanochannel. (a) Non-methylated and methylated T4 DNA molecules were digested by using the restriction enzyme Sall. Methylated DNA molecules showed a resistance to enzymatic digestion. (b) The contraction process of a single DNA molecule was observed with the inverted fluorescence microscope after switching off the electric field. The florescence images of the contraction process for a non-methylated and a methylated single T4 DNA molecule inside the nanochannel. Scale bars are 10 μm. (c) Data points for average length of non-methylated (blue) and methylated (red) single T4 DNA molecules inside the nanochannel plotted as a function of time. Red and blue dotted lines showed fitting curves from Equation (1). Error bars show the standard deviation for a series of measurements (*n* = 5).

Methylation of DNA molecules causes a difference in their contraction process in the nanochannel (Figure 3). The presence of methylated DNA molecules was confirmed by enzymatic digestion; the methylated DNA molecules had one band, while the non-methylated DNA molecules had several bands (Figure 3(a)). After single DNA molecules were introduced into and elongated in the nanochannel by applying the electric field (Figure 3(b)), the time-course contraction process of a single methylated and a single non-methylated DNA molecule in the nanochannel was obtained as shown in Figure 3(c). We found that the length of the methylated DNA molecule after a 7-s contraction was longer than the length of the non-methylated one. And the data points of DNA length showed the contraction rate of the methylated DNA molecule was slower than that of the non-methylated one (Figure 3(c)). Since the exact contraction mechanism of methylated DNA molecules in the nanochannel must be rather complex, such DNA contraction behaviors are an interesting subject for theoreticians and experimentalists alike.

To interpret the difference in the contraction process, we focused on the rigidity increase by methylation. It has been reported that the rigidity of DNA molecules increases in response to DNA methylation.[9–11] Conformational spaces restricted by steric hindrance of the bulky methyl groups trigger an increased persistence length of methylated DNA molecules of about 30% with a methylation rate of 20% compared to non-methylated DNA molecules.[12–14] In the present study, we methylated all cytosine residues (C^5^) on 4816 CpG positions within the DNA molecules, which have 31,034 cytosine in total, using CpG methyltransferase; the methylation rate of DNA molecules was about 15%, and we could calculate a 23% increase of persistence length to get the theoretical value.[10] The DNA lengths after the 7-s contraction of non-methylated and methylated DNA molecules were 11.06 μm and 13.58 μm, respectively (Figure 3(c)); this was a 23% increase in the contracted length, which was the same per cent increase as the theoretical increase of the persistence length with a methylation rate of 15%. The contracted length of DNA molecules has been reported to have a relationship with the persistence length,[15] and therefore our explanation of the contracted length increasing using persistence length makes sense.

Seven-second contraction of non-methylated and 15% cytosine-methylated (100% CpG-methylated) DNA molecules showed a significant difference of the contracted length around 2.5 μm. Since the spatial resolution of optical microscopy is limited to *c*.200 nm,[16] we might find a significant difference of the contracted length between non-methylated and 8% CpG-methylated DNA molecules. And even if we use stimulated emission depletion condition with optical resolution of 70 nm,[17] we might distinguish the contracted length of non-methylated from 2% CpG-methylated DNA molecules. The report for methylation frequencies in human malignancies shows that a possibility for 8% methylation discrimination is good enough for various cancers prediction in their early stage.[5] This kind of prediction has enormous significance in using DNA molecules with at least 100 kbp due to an importance of ensuring genome information integrity.[18,19]

In order to explain the difference in the contraction process of methylated and non-methylated single DNA molecules, it can be plotted using the following equation [20]:

(1) $$l\left(t\right)=l_{e}+\left(l_{0}-l_{e}\right)\exp\left(-\frac{5k_{B}TD^{1/3}}{8\pi \eta L^{2}{\left(pw\right)}^{2/3}}t\right)$$

where *l*_*e*_ and *l*_0_ are the equilibrium length and the initial length in the nanochannel, respectively, *η* is the viscosity of the solvent, *L* is DNA contour length, *p* is the DNA persistence length, *w* is the DNA molecule width, *k*_*B*_ is the Boltzmann constant, and *D* is channel diameter. Using fitting curves based on the above equation, we calculated the coefficients in the exponential expression as 0.303 and 0.348 s^−1^ for methylated and non-methylated DNA molecules, respectively. An inverse of the coefficients in the exponential expression showed relaxation times for methylated and non-methylated DNA molecules of 3.300 and 2.874 s, respectively. From the fitted and calculated relaxation time, we also calculated a 23% increase for persistence length, and these results highlighted that the contraction rate of methylated DNA molecules was slower than that of non-methylated DNA molecules in the nanochannel; and therefore, our methodology could distinguish methylated and non-methylated DNA molecules based on a difference of the relaxation time related to a difference of the persistence length.

Since methylation of DNA molecules changes the contracted length and the contraction process, the fluorescence intensity profile of DNA molecules may be able to show methylation positions. Considering methylation causes relatively long contracted length and slow contraction process, we suppose that fluorescence intensity profiles reveal methylation location of DNA molecules, approximately. This kind of measurement allows researchers to identify approximate methylation location and distinguish methylated or non-methylated DNA molecules. As for cancer prediction in early stages, detecting methylated rate increase abnormally is more important than reading out sequence information, and therefore our proposed measurement would contribute to first screening of DNA methylation analysis.

## 4. Conclusions

In summary, we demonstrated that our nanochannel device is a powerful tool to detect DNA methylation without any protein modification at the level of a single DNA molecule. It assists in detecting DNA methylation with a small amount of sample and in a relatively short time including pretreatment and analysis (approximately 2 h). We use the contracted length and contraction rate of a single DNA molecule to determine whether or not a DNA molecule is methylated, and identify methylation location of DNA molecules, approximately. Our methodology allows users to analyze intact DNA molecules in nanochannels. We expect that our device will become an important part of biomolecule analysis systems.

## Competing financial interests

The authors declare no competing financial interests.

## Funding: 

This research was supported by the Cabinet Office, Government of Japan and the Japan Society for the Promotion of Science (JSPS) through the Funding Program for World-Leading Innovative R&D on Science and Technology (FIRST Program), the ImPACT Program of the Council for Science, Technology and Innovation (Cabinet Office, Government of Japan), Nanotechnology Platform Program (Molecule and Material Synthesis) of the Ministry of Education, Culture, Sports, Science and Technology (MEXT), Japan, the JSPS Grant-in-Aid for Scientific Research (A) [16H02091], and the Grant-in-Aid for Scientific Research on Innovative Areas ‘Nanomedicine Molecular Science’ [number 26107709] from the MEXT and PRESTO, Japan Science and Technology Agency (JST).
